# The influence mechanism of source experience of the knowledge on the knowledge transfer performance: The role of political skill and knowledge barriers

**DOI:** 10.3389/fpsyg.2022.980453

**Published:** 2022-10-12

**Authors:** Shih-Liang Lee, Tsang-Kai Hung, Mu Tian

**Affiliations:** ^1^Human Resource Management, National Changhua University of Education, Changhua City, Taiwan; ^2^School of Business, Ningbo Tech University, Ningbo, Zhejiang, China

**Keywords:** characteristics of the source of knowledge, political skill, knowledge barriers, knowledge transfer performance, knowledge source

## Abstract

Exploring the relationship between characteristics of the source of knowledge and knowledge transfer performance seems to be crucial in order to make up for the lack of research on the political skills of knowledge sources in the process of knowledge transfer. For this reason, this study conducts a paired-sample questionnaire survey to achieve the research purpose. One direct supervisor was paired with 1∼4 subordinates; 274 other-reported questionnaires were sent out to supervisors and 1,096 self-reported questionnaires to subordinates. A total of 214 valid supervisor questionnaires and 630 valid subordinate questionnaires were collected. The finding demonstrates that knowledge sources with political skills can reduce knowledge barriers to knowledge transfer as well as affect knowledge transfer performance. This research presents a valid model that comprises the antecedents (characteristics of the knowledge source), mediators (knowledge barriers), moderators (political skill), and consequences of knowledge transfer performance of firms. Moreover, this study provides several meaningful directions for future research.

## Introduction

Knowledge is the most strategic resource of an organization, and it is beneficial to maintaining organization’s sustainable competitive advantage (Martinez-Sanchez et al., 2021; Rosiello and Maleki, 2021; Oh and Lee, 2022). Knowledge also can enhance the discovery and exploitation of opportunities (Wiklund and Shepherd, 2003), and it can be used as a source for generating and promoting innovative activities (Amoroso et al., 2018; Lei et al., 2021). Broadly, knowledge is categorized into two different types: explicit knowledge and tacit knowledge (Nonaka and Takeuchi, 1995). Explicit knowledge is easy to formalize, manage and transfer, whereas, tacit knowledge is more complex and difficult to manage (Faccin et al., 2019).

According to the knowledge-based view (Grant, 1996), a firm’s existing knowledge base delimits its scope and capacity to comprehend and apply novel knowledge to innovations (Hill and Rothaermel, 2003). In order to promote innovation performance of an organization, effective knowledge transfer is essential (Khoirunnisa and Almahendra, 2022; Wang et al., 2022). Nonaka and Takeuchi (1995) pointed out that the transfer of knowledge from one individual to another is an important topic in knowledge management. Ritala and Stefan (2021) suggested that knowledge sharing and transfer primarily occur between individuals, resulting in organizational-level implications.

According to the knowledge-based view, tacit knowledge is abstract and can only be transmitted through the active involvement of the source of knowledge (Dhanaraj et al., 2004). Tacit knowledge has ambiguity or stickiness transfer barriers. If the interpersonal relationship and communication between the knowledge source and recipient cannot be used (Nonaka and Takeuchi, 1995), it will be difficult to duplicate and transfer (Bierly et al., 2009). For this reason, the successful interaction between the knowledge source and the recipient depends on the quality of the relationship (Burmeister et al., 2020, 2022).

Previous research has concluded that knowledge transfer is affected by four groups: knowledge characteristics, source characteristics and recipient characteristics, relationship between source and recipient, and knowledge integration mechanisms (Junni et al., 2012, 2015; Ciabuschi et al., 2017). The effectiveness of knowledge transfer can be measured by changes in knowledge and performance (Liu, 2018). However, how the variables in the relationship between knowledge source and knowledge recipient affect the effectiveness of knowledge transfer are not explored in existing literatures. Therefore, this research asks the following question: What is the relationship between the characteristics of the knowledge source and the performance of knowledge transfer?

In addition, if knowledge is not adsorptive by the recipient, it is not considered as a true transfer success. The concept of political skill originated from Pfeffer (1981) and Mintzberg (1983), who considered that an organization is like a political environment. When an individual fight alone, it is difficult to succeed. Using specific social skills can enable the individual to achieve success in the organization. A close relationship also depends on mutual trust, promise, and frequent communication (Sikombe and Phiri, 2019). The arduous relationship between each other will adversely affect the transfer of knowledge (Singh, 2019). Therefore, those with political skills can promote interpersonal interactions and effect job performance through personal influence (Semadar et al., 2006; Liu et al., 2007). However, previous literature on knowledge transfer processes lacks a discussion of the relationship between political skills and knowledge sources.

**FIGURE 1:**
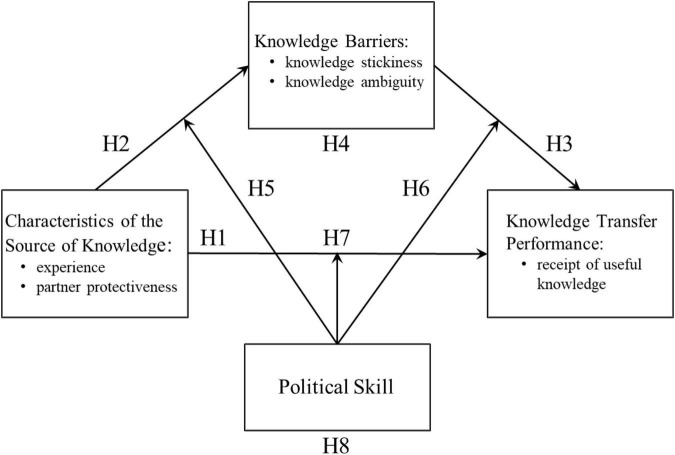
Research structure.

Therefore, the purpose of this research is to explore the relationship between characteristics of the source of knowledge and knowledge transfer performance, and reveal the role of political skills of knowledge sources in the process of knowledge transfer. The structure of this research is shown in Figure 1. The study offers three contributions. First, this research confirms that the political skills of the knowledge source have an effect of moderation on the relationship between the characteristics of the knowledge source and the knowledge barriers.

The political skills of the knowledge source also have an effect of moderation on the relationship between the knowledge barriers and the performance of knowledge transfer. Second, the political skills of the knowledge source have an effect of moderation on the relationship between the characteristics of the knowledge source and the performance of knowledge transfer. Finally, the political skills of the knowledge source also produce moderated mediation on the relationship among knowledge source, knowledge barriers, and knowledge transfer performance.

## Literature review and hypotheses

Polanyi (1962) divided knowledge into explicit knowledge and tacit knowledge according to whether knowledge can be clearly expressed and effectively transferred.

Tacit knowledge refers to knowledge that is non-verbal, intuitive, and unable to be described in detail. Explicit knowledge refers to knowledge that can be formalized, institutionalized, and conveyed in words. Explicit knowledge is easy to formalize, manage and transfer, whereas tacit knowledge is more complex and difficult to manage (Nonaka and Takeuchi, 1995).

Knowledge transfer performance can be measured in terms of the recipient’s receipt of useful knowledge (Levin and Cross, 2004; Evans et al., 2019). Previous research has demonstrated the influence of the characteristics of the source of knowledge, such as the source’s experience (Olvera and Avellaneda, 2019; Honoré, 2022) or source’s partner protectiveness (Ho and Wang, 2015) on knowledge transfer performance.

Political skill is defined as “the ability to effectively understand others at work, and to use such knowledge to influence others to act in ways that enhance one’s personal and/or organizational objectives” (Ferris et al., 2005). Numerous studies have shown that employees with political skills have fewer stressors in the workplace, are more productive, and have higher rates of career success (Summers et al., 2020; Sun, 2022) because politically skilled individuals may realize that their resources can be significantly enhanced by engaging in beneficial alliances and interpersonal interactions (Ain et al., 2022). Correspondingly, politically-skilled individuals can avoid hiding knowledge because they perceive relationships as a resource worth maintaining through active networking and knowledge-sharing in highly politicized contexts (De Clercq et al., 2020).

As for knowledge barriers, in line with the concept of sticky information (Von Hippel, 1994), Szulanski (1996) categorized the barriers of knowledge as four constructs as follows: barriers inherent to the content of knowledge; barriers inherent to the context; barriers inherent to the source; and barriers inherent to the recipient. This barrier can be found at activities related to innovation, the degree of transfer, the use of superior knowledge (de Araújo et al., 2020).

Empirical research has demonstrated that tacit knowledge has ambiguity or stickiness transfer barriers (Sheng et al., 2013). Current scholars generally use three research modes, channel, conversion, and embeddedness, to solve the barriers to knowledge transfer. The channel model advocates that organizations only need to remove barriers (including environmental barriers and transmission barriers) in the knowledge transfer process in order to ensure the effective knowledge transfer (Sheng et al., 2013; Ciabuschi et al., 2017).

Meanwhile, Ikujiro Non-aka first proposed the knowledge conversion model, claiming that organizations should provide a mechanism for knowledge conversion. Only when knowledge is properly converted can knowledge be effectively transferred. The process of knowledge conversion is actually the process of knowledge creation (Nonaka, 1994, 2009; Nonaka and Takeuchi, 1995; Alavi and Leidner, 2001). Finally, the embeddedness model advocates that organizations and other organizations are systematically engaged in interactive learning through an embeddedness institutional environment. Only through interpersonal interaction and participation in the daily work of the community can organizational members effectively transfer knowledge (Pousa and Mathieu, 2015).

### Relationship between the characteristics of the source of knowledge and the performance of knowledge transfer

According to the knowledge-based view, knowledge is the most strategic resource of an organization, and it is the source of an organization’s competitive advantage (Teece et al., 1997). External sources of knowledge creation include suppliers, distributors, research units, universities, government projects, and market stakeholders (Amoroso et al., 2018; Iftikhar and Ahola, 2022). The core concept of resource dependence theory is that an organization imports elements such as people and raw materials from the external environment, then undergoes internal transformation before exporting products or services to the environment to obtain more resources and continue the exchange process (Pfeffer, 1972; Pfeffer and Salancik, 1978). It is important to determine which variables in this exchange process are the key factors affecting the effectiveness of knowledge transfer. Previous research has concluded that knowledge transfer is affected by four groups: knowledge characteristics, source characteristics, and recipient characteristics, relationship between source and recipient, and knowledge integration mechanisms (Junni et al., 2012, 2015; Ciabuschi et al., 2017).

Nonaka and Takeuchi (1995) pointed out that the transfer of knowledge from one individual to another is an important topic in knowledge management. Knowledge transfer is the transfer of experience to another organization through an experienced unit, such as an individual, a group, or a department—that is, the process by which the knowledge recipient obtains knowledge from the source (Liao and Hu, 2007). The research of Simonin (1999a) pointed out that the characteristics of partner protectiveness of the knowledge source with a higher degree will make the knowledge recipient unable to clearly understand the consequences of knowledge transfer, which will easily cause knowledge ambiguity and hinder knowledge transfer performance. In addition, Levin and Cross (2004) and Evans et al. (2019) stated that knowledge transfer performance can be measured against the recipient receipt of useful knowledge. Based on these research viewpoints, this research proposes the following hypotheses:

H1a: The experience of the knowledge source has a positive effect on the recipient’s receipt of useful knowledge.

H1b: The partner’s protectiveness of the knowledge source has a negative effect on the recipient’s receipt of useful knowledge.

### Relationship among characteristics of knowledge sources, knowledge barriers, and knowledge transfer performance

Empirical research has demonstrated that tacit knowledge has ambiguity or stickiness transfer barriers (Sheng et al., 2013). If the interpersonal relationship and communication between the knowledge source and recipient cannot be used (Nonaka and Takeuchi, 1995; Summers et al., 2020; Munyon et al., 2021), it will be difficult to duplicate and transfer (Bierly et al., 2009). The transfer of tacit knowledge requires teaching and the willingness of the knowledge source to fully cooperate. This means that the degree of partner protectiveness must be minimized. Even if the knowledge source has a rich experience, if the knowledge source is unwilling to share knowledge with others, the knowledge obtained by the knowledge recipient will be incomplete knowledge. The knowledge recipient cannot fully understand the connotation of the new knowledge, which in turn affects the performance of knowledge transfer (Burmeister et al., 2020). Based on these research viewpoints, the following hypotheses are proposed:

H2a: The experience of the knowledge source has a negative effect on knowledge stickiness.

H2b: The experience of the knowledge source has a negative effect on knowledge ambiguity.

H2c: The partner’s protectiveness of the knowledge source has a positive effect on knowledge stickiness.

H2d: The partner’s protectiveness of the knowledge source has a positive effect on knowledge ambiguity.

Simonin (1999a,b) also pointed out that knowledge ambiguity is an antecedent variable that reduces the performance of knowledge transfer. Tacit knowledge has ambiguity or stickiness transfer barriers (Muniz et al., 2022; Perumal and Sreekumaran Nair, 2022). Tacit knowledge is linearly related to its immovability, causing barriers to knowledge transfer (Reed and DeFillippi, 1990). The concept of knowledge stickiness comes from Von Hippel’s (1994) sticky information, which is used to describe difficult-to-deliver information. Szulanski (1996) proposed that knowledge stickiness refers to the obstacles encountered in the process of knowledge transfer, that is, the difficulty of knowledge transfer within an organization. Knowledge stickiness can hinder the transfer of knowledge from Source of Knowledge to recipient (Sheng et al., 2013; Sikombe and Phiri, 2019). Based on these research findings, this research proposes the following hypotheses:

H3a: Knowledge stickiness has a negative effect on the recipient’s receipt of useful knowledge.

H3b: Knowledge ambiguity has a negative effect on the recipient’s receipt of useful knowledge.

Simonin (1999a,b) research on strategic alliance partners identified seven factors that affect the degree of knowledge ambiguity; among them, knowledge related includes tackiness, specificity, and complexity. In addition, the gap between knowledge source and recipient includes cultural distance and organizational distance (Ciabuschi et al., 2017). When an individual encounters a job with high ambiguity, it is difficult to solve the problem using individual knowledge. Through the transfer of knowledge among members of the organization, the problem-solving ability of the individual and the organization can be improved (Alexander et al., 2020). The previous research channel model advocates that organizations only need to remove barriers (including environmental barriers and transmission barriers) in the knowledge transfer process in order to ensure effective knowledge transfer (Ciabuschi et al., 2017; Sikombe and Phiri, 2019; Ouakouak et al., 2021). Based on these research findings, this research proposes the following hypotheses:

H4a: Knowledge stickiness mediates the relationship between the experience of the knowledge source and the recipient’s receipt of useful knowledge.

H4b: Knowledge ambiguity mediates the relationship between the experience of the knowledge source and the recipient’s receipt of useful knowledge.

H4c: Knowledge stickiness mediates the relationship between the partner’s protectiveness of the knowledge source and the recipient’s receipt of useful knowledge.

H4d: Knowledge ambiguity mediates the relationship between the partner’s protectiveness of the knowledge source and the recipient’s receipt of useful knowledge.

### Relationship among political skills, characteristics of knowledge sources, and knowledge transfer performance

The concept of political skill originated from Pfeffer (1981) and Mintzberg (1983), who asserted that an organization is like a political environment. When an individual fights alone, it is difficult to succeed; conversely, when an individual use specific social skill, the individual has a better chance of achieving success in the organization. Mintzberg (1983) further defined this specific social skill as a political skill—that is, the ability to influence others through the use of influential strategies. Harris et al. (2007) pointed out that people with better political skills can obtain a more positive image in the impression of others, and political skills will strengthen the positive relationship between impression management behavior and job performance. Political skills can provide the ability to understand others, and those with political skills know how to show sincerity, social interaction, interpersonal interaction, and impression management in work situations, adjusting their behaviors according to different situations in order to influence others appropriately and effectively (Semadar et al., 2006).

The arduous relationship between the knowledge source and recipient will have an adverse effect on knowledge transfer (Baum and Ingram, 1998; Akram et al., 2020), and barriers to knowledge stickiness will increase (Schuller, 2014; Sikombe and Phiri, 2019). The successful interaction between the knowledge source and the recipient depends on the quality of the relationship. A close relationship also depends on mutual trust, promise, and frequent communication (Sikombe and Phiri, 2019). The arduous relationship will also adversely affect knowledge transfer (Szulanski, 1996). The previous research on the embeddedness model of knowledge transfer barriers advocates that only through interpersonal interaction and participation in the daily work of the community can organizational members effectively transfer knowledge (Pousa and Mathieu, 2015; Nguyen et al., 2021; Bari et al., 2022). Based on these research viewpoints, this research proposes the following hypotheses:

H5a: Political skills of the knowledge source have a moderating effect on the relationship between experience and knowledge stickiness.

H5b: Political skills of the knowledge source have a moderating effect on the relationship between experience and knowledge ambiguity.

H5c: Political skills of the knowledge source have a moderating effect on the relationship between partner protectiveness and knowledge stickiness.

H5d: Political skills of the knowledge source have a moderating effect on the relationship between partner protectiveness and knowledge ambiguity.

Szulanski (1996) stated that knowledge stickiness would hinder the transfer of knowledge from the source to the recipient. Simonin (1999a,b) also pointed out that knowledge ambiguity is an antecedent variable that reduces the performance of knowledge transfer. If the knowledge source encounters difficulties in expressing the process of transferring tacit knowledge, it can increase the cooperation time with the recipient and overcome the difficulty of expressing tacit knowledge through regular interaction in the work situation (Collins and Hitt, 2006). Those with political skills can demonstrate appropriate behaviors in different work situations through personal influence, promote interpersonal interaction at work, and generate effectiveness (Liu et al., 2007).

The previous research on the embeddedness model of knowledge transfer barriers advocates that only through interpersonal interaction and participation in the daily work of the community can organizational members effectively transfer knowledge (Pousa and Mathieu, 2015). Levin and Cross (2004) and Evans et al. (2019) pointed out that knowledge transfer performance can be measured by the receipt of useful knowledge by the knowledge recipient. Based on such research findings, this research proposes the following hypotheses:

H6a: Political skills of the knowledge source have a moderating effect on the relationship between knowledge stickiness and receipt of useful knowledge.

H6b: Political skills of the knowledge source have a moderating effect on the relationship between knowledge ambiguity and receipt of useful knowledge.

In terms of the key factors affecting the effectiveness of knowledge transfer, previous studies have explored the impact of the characteristics of the knowledge source on the performance of knowledge transfer (Simonin, 1999a,b; Amoroso et al., 2018). The process of knowledge transfer requires a strong relationship between the source of knowledge and the recipient as well as a climate of mutual affinity, mutual respect, and trust (Junni and Sarala, 2011). Political skills have the ability to understand others in a work situation. Through the use of this ability, they can influence others and achieve personal or organizational goals (Ahearn et al., 2004). Political skills involve interaction in the workplace and are suitable for using this ability to understand others and further influence others in a work situation (Harris et al., 2007). Individuals with better political skills know how to show behavior in a way that seems sincere and hides self-interested motives, ultimately achieving the purpose of influencing others (Ferris et al., 2005).

H7a: Political skills of the knowledge source have a moderating effect on the relationship between experience and the receipt of useful knowledge.

H7b: Political skills of the knowledge source have a moderating effect on the relationship between partner protectiveness and the receipt of useful knowledge.

H8a: The political skills of the knowledge source mediate the relationship among experience, knowledge stickiness, and receipt of useful knowledge.

H8b: The political skills of the knowledge source mediate the relationship among experience, knowledge ambiguity, and the receipt of useful knowledge.

H8c: The political skills of the knowledge source mediate for the relationship among partner protectiveness, knowledge stickiness, and receipt of useful knowledge.

H8d: The political skills of the knowledge source mediate the relationship among partner protectiveness, knowledge ambiguity, and receipt of useful knowledge.

## Methodology

This research used purposive sampling to collect data. The data collection covered whether different educational levels in the organization affect the relationship between the characteristics of the source of knowledge and the performance of knowledge transfer. The sample included the supervisors and employees of private enterprises as well as the public and private sectors. The research objective did not limit the industry or job attributes. For this study, the questionnaires were distributed by mail. Each set was accompanied by a questionnaire description letter, a formal questionnaire, a return envelope, and a letter that could be completed to answer the questionnaire. This reduced respondents’ unnecessary anticipation and work-related troubles.

The test was administered in an anonymous manner. After collecting the questionnaire responses, data sorting, coding, and file creation were carried out. Statistical software LISREL 8.7, SPSS 22, JASP.14.11, and PROCESS 3.3 were used for the data analysis. LISREL 8.7 was used for a confirmatory factor analysis (CFA), followed by SPSS 22 for descriptive statistics and a correlation analysis. JASP.14.11 was then used to analyze the mediated effect. Finally, PROCESS 3.3 was used to analyze the moderated and moderated mediation effects.

According to Hinkin’s (1998) research, in order to avoid the common method variance (CMV) problem, it is necessary to answer all measurement questions in the scale using multiple sources of subjects. Measures were both self-reported (subordinate) and other-reported (supervisor). The questionnaire survey adopted the matching method, and the employees and supervisors used the matching method to complete the answers; the color and code were matched in advance. Based on the principle of one direct supervisor being responsible for 1–4 subordinates, 250 sets of supervisor and subordinate matching questionnaires were expected to be issued.

The questionnaire was divided into the supervisor questionnaire (other-reported) and subordinate questionnaire (self-reported). Supervisors and subordinates were asked to provide basic personal information. The supervisors provided information on the knowledge transfer performance of the subordinates (other-reported); the subordinates answered questions about the characteristics of the knowledge source, political skills, knowledge barriers, and knowledge transfer performance (self-reported).

The reasons for adopting scales in this study are as follows: (1) The independent variables adopt the experience scale and partner protectiveness scale of Simonin (1999a,b). The reason is that experience is considered to be one of the good predictors of job performance (Moscoso and Iglesias, 2009). In addition, Szulanski (1996) found that the characteristic of partner protectiveness of knowledge sources is the source of barriers to knowledge transfer. (2) The mediator variable adopts the knowledge stickiness scale of Jensen and Szulanski (2004) and the knowledge ambiguity scale of Simonin (1999a). The main reason is that tacitness of knowledge has ambiguity or stickiness transfer barriers (Nonaka and Takeuchi, 1995). (3) The moderator variable adopts the political skill scale of Perreweì et al. (2004). The reason is that political skills have the ability to adjust their behavior according to different situations in order to influence others appropriately and effectively (Semadar et al., 2006). (4) The dependent variable adopts the scale of receipt of useful knowledge by Levin and Cross (2004) and Evans et al. (2019). Because Levin and Cross (2004) and Evans et al. (2019) stated that knowledge transfer performance can be measured against the recipient receipt of useful knowledge.

Previous research pointed out, the variables in the relationship between knowledge source and knowledge recipient that will affect the effectiveness of knowledge transfer are very and worthy of further discussion (Junni et al., 2012, 2015; Ciabuschi et al., 2017).

## Data analysis and results

A total of 274 supervisor questionnaires (other-reported) and 1,096 subordinate questionnaires (self-reported) were issued, and 217 supervisor questionnaires (other-reported) and 805 subordinate questionnaires (self-reported) were collected. After checking the sample data, those supervisors and subordinates who did not have experience with knowledge transfer or interaction with the knowledge source as well as invalid samples were excluded. The actual available sample data came from 214 supervisor questionnaires (other-reported) and 630 subordinate questionnaires (self-reported).

The effective response rate of subordinate questionnaires was 57.48%. According to the statistical analysis of the characteristics of the recovered samples, 47.9% of the employees were male and 52.1% were female; 7.3% were younger than 25 years old, 21.1% were 26–30 years old, 41.1% were 31–40 years old, 23.0% were 41–50 years old, and 7.5% were older than 51 years old. Employees with high school education or below accounted for 13.8% of the sample, university accounted for 65.7%, and graduate school or above accounted for 20.5%.

### Correlation analysis

A correlation analysis was used to measure the strength of the relationship between variables and confirm the common variation relationship of each variable (Table 1). (1) Both experience and receipt of useful knowledge (self-reported: *r* = 0.52, *p* < 0.01; other-reported: *r* = 0.24, *p* < 0.01) showed a significant positive correlation. (2) Neither partner protectiveness and receipt of useful knowledge (self-reported: *r* = 0.07, *p* = 0.06; other-reported: *r* = 0.04, *p* = 0.0307) showed no significant correlation. (3) Experience and knowledge stickiness (*r* = –0.15, *p* < 0.01) showed a significant negative correlation, while experience and knowledge ambiguity (*r* = 0.27, *p* < 0.01) showed a significant positive correlation. (4) Partner protectiveness and knowledge stickiness (*r* = 0.32, *p* < 0.01) had a significant positive correlation; partner protection and knowledge ambiguity (*r* = 0.00, *p* = 0.937) did not have a significant correlation. (5) Knowledge stickiness and receipt of useful knowledge (self-reported: *r* = –0.09, *p* < 0.05; other-reported: *r* = –0.09, *p* < 0.05) showed a significant negative correlation. (6) Knowledge ambiguity and receipt of useful knowledge (self-reported: *r* = 0.51, *p* < 0.01; other-reported: *r* = 0.20, *p* < 0.01) demonstrated a significant positive correlation.

**TABLE 1:** Mean, standard deviation, and correlations.

	*M*	SD	1	2	3	4	5	6	7	8
1. Education level^a^	2.56	0.58								
2. Experience	3.92	0.69	–0.05	(0.88)						
3. Partner protectiveness	3.22	0.90	–0.29	0.13**	(0.69)					
4. Political skill	3.56	0.64	−0.09*	0.46**	0.05	(0.89)				
5. Knowledge stickiness	2.82	0.67	−0.09*	−0.15**	0.32**	0.03	(0.79)			
6. Knowledge ambiguity	3.22	0.74	−0.13**	0.27**	0.00	0.48**	0.09*	(0.65)		
7. RUK (SR)	3.70	0.61	−0.08*	0.52**	0.07	0.53**	−0.09*	0.51**	(0.91)	
8. RUK (OR)	3.79	0.56	–0.01	0.24**	0.04	0.25**	−0.09*	0.20**	0.32**	(0.89)

*N* = 630, *p < 0.05, **p < 0.01.

The scale reliability value Cronbach’s α is in brackets.

^*a*^High school or below = 1, university = 2, graduate school or above = 3.

RUK, receipt of useful knowledge; SR, self-reported; OR, other-reported.

### Confirmatory factor analysis

The factor loading value of each item in the confirmatory factor analysis of this study ranged between 0.49 and 0.76, meeting Hair et al.’s (2014) standard that the absolute value of factor loading should be greater than 0.30. The composite reliability (CR) was 0.94, which is in line with Fornell and Lacker’s (1981) recommendation to reach above 0.60. The average variance extracted (AVE) of 0.36 barely met Fornell and Lacker’s (1981) accepted standard. The χ^2^/*df* value was 2.7, meeting the standard of less than five as recommended by Hooper et al. (2008). The GFI value was 0.90, which also met Hooper et al.’s (2008) standard that the value should be greater than 0.90. The NNFI value was 0.97, which met Bentler and Bonnet’s (1980) recommendation that the value should be greater than 0.09.

The IFI value was 0.97, which is in line with Bollen’s (1989) suggestion that the value should be greater than 0.90. The CFI value was 0.97, which complied with the standard recommended by Hu and Bentler (1999) that the value should be greater than 0.90. The SRMR value was 0.05, which also met Hair et al.’s (2014) recommendation that the value should be less than 0.08. The RMSEA value was 0.05, which was also in line with Hair et al.’s (2014) recommendation that the value should be less than 0.08. Thus, all the verifications and analyses of the overall model fit were up to the standards recommended by scholars.

### Mediating effect test of knowledge barriers

We tested knowledge barriers’ mediation of the relationship between the characteristics of the knowledge source and the performance of knowledge transfer (see Table 2). First, knowledge stickiness showed an indirect effect between experience and receipt of useful knowledge (self-report) (EX→KS→RUK[SR]) (*B* = 0.02, *p* < 0.01), indicating a significant positive forecasting effect. According to bootstrapping, the 95% confidence interval (CI) test (0.005, 0.039) did not contain zero. In addition, knowledge stickiness demonstrated an indirect effect between experience and receipt of useful knowledge (other-report) (EX→KS→RUK[OR]) (*B* = 0.02, *p* < 0.05), which also indicated a significant positive forecasting effect. The bootstrapping 95% CI test indicated that it did not contain zero (0.003, 0.042). Therefore, the value of the indirect effect presented through this verification method was significant. The results support H4a.

**TABLE 2:** Knowledge barriers mediator the indirect effects between the characteristics of knowledge sources and the performance of knowledge transfer.

Indirect effect	*B*	SE	*z*	*p*	95% CI	
					
					Lower	Upper
EX→KS→RUK (SR)	0.02**	0.00	2.606	0.009	0.005	0.039
EX→KA→RUK (SR)	0.11***	0.01	6.251	<0.001	0.077	0.154
PP→KS→RUK (SR)	−0.03**	0.01	–2.8	0.005	–0.060	–0.010
PP→KA→RUK (SR)	–0.01	0.01	–0.964	0.335	–0.049	0.019
EX→KS→RUK (OR)	0.02*	0.01	2.11	0.035	0.003	0.042
EX→KA→UK (OR)	0.04***	0.01	3.591	<0.001	0.023	0.076
PP→KS→RUK (OR)	−0.03*	0.01	–2.209	0.027	–0.064	–0.005
PP→KA→RUK (OR)	–0.00	0.00	–0.941	0.347	–0.021	0.007

*N* = 630, *p < 0.05, **p < 0.01, ***p < 0.001.

Five thousand sets of samples are repeatedly selected for interval estimation under the 95% confidence interval of bootstrapping.

CI, confidence interval; EX, experience; PP, partner protectiveness; KS, knowledge stickiness; KA, knowledge ambiguity; RUK, receipt of useful knowledge; SR, self-report; OR, other-report.

Second, knowledge ambiguity had an indirect effect between experience and receipt of useful knowledge (self-report) (EX→KA→RUK[SR]) (*B* = 0.11, *p* < 0.001), with a significant positive forecasting effect. The bootstrapping 95% CI test produced no zero (0.077, 0.154). In addition, knowledge ambiguity had an indirect effect between experience and receipt of useful knowledge (other-report) (EX→KA→RUK[OR]) (*B* = 0.04, *p* < 0.001), showing a significant positive forecasting effect. The bootstrapping 95% CI test showed no zero (0.023, 0.076). Therefore, the value of the indirect effect shown by these verification methods was significant, and the results support H4b.

Third, knowledge stickiness had an indirect effect between partner protectiveness and receipt of useful knowledge (self-report) (PP→KS→RUK[SR]) (*B* = –0.03, *p* < 0.01), showing a significant negative forecasting effect. The bootstrapping 95% CI test did not include zero (–0.060, –0.010). Meanwhile, shows that knowledge stickiness had an indirect effect between partner protectiveness and receipt of useful knowledge (other-report) (PP→KS→RUK[OR]) (*B* = –0.03, *p* < 0.05), demonstrating a significant negative forecasting effect. The bootstrapping 95% CI test showed no zero (–0.064, –0.005). Therefore, the value of the indirect effect shown by this verification method was significant, and the results support H4c.

Fourth, knowledge ambiguity did not have an indirect effect between partner protectiveness and receipt of useful knowledge (self-report) (PP→KA→RUK[SR]) (*B* = –0.01, *p* = 0.335), which was not significant. The bootstrapping 95% CI test contained zero (–0.049, 0.019). Moreover, knowledge ambiguity did not have an indirect effect between partner protectiveness and receipt of useful knowledge (other-report) (PP→KA→RUK[OR]) (*B* = –0.00, *p* = 0.347), which was not significant. The bootstrapping 95% CI test contained zero (–0.021, 0.007). Therefore, the value of the indirect effect presented by the above verification method was not significant, and the results of this research do not support H4d.

### Moderating effect test of political skill

**FIGURE 2:**
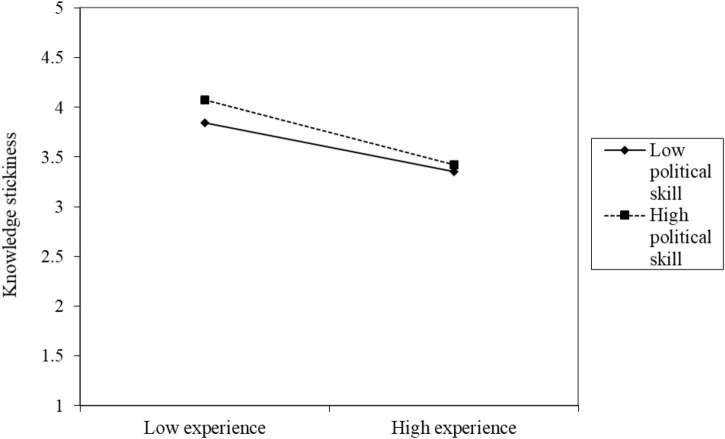
Diagram of the interaction of political skills affecting experience on knowledge stickiness.

We tested moderating effect test of political skill. Table 3 presents the self-reported results while Table 4 summarizes the other-reported results. First, in model M1, the interaction between the independent variable experience and the moderation variable political skills (EX × PS) on the mediation variable knowledge stickiness (KS) had significant predictive power in both self-reported (B = –0.08, *p* < 0.05) and other-reported (B = –0.15, *p* < 0.05) results. These results support H5a. Figure 2 shows that high experience has lower knowledge stickiness whereas low experience has higher knowledge stickiness. High political skills have a stronger moderating effect than low political skills.

**TABLE 3:** Analysis of the moderating effect of political skills to experience on receiving useful knowledge (self-report).

Predictive variable	Mediation: KS		Mediation: KA		Dependent: RUK (SR)	
			
	M1		M2		M3	
			
	*B*	SE	*B*	SE	*B*	SE
Constant	0.35*	0.14	0.31*	0.12	3.71***	0.06
Control education^a^	–0.15	0.06	−0.14*	0.06	–0.00	0.03
Independent EX	−0.24***	0.04	0.05	0.04	0.17***	0.02
Moderation PS	0.14***	0.04	0.45***	0.04	0.16***	0.02
Interaction EX × PS	−0.08*	0.06	–0.01	0.02	−0.03*	0.01
Mediation						
KS					−0.08***	0.01
KA					0.18***	0.02
Interaction						
KS × PS					0.04*	0.01
KA × PS					0.04	0.01
*R* ^2^	0.243		0.245		0.488	
*F*-test	9.8398***		50.6931***		70.039***	

*N* = 630, *p < 0.05, ***p < 0.001.

^*a*^High school or below = 1, university = 2, graduate school or above = 3.

EX, experience; PS, political skill; KS, knowledge stickiness; KA, knowledge ambiguity; RUK, receipt of useful knowledge; SR, self-report.

**TABLE 4:** Analysis of the moderating effect of political skills to experience on receiving useful knowledge (other-report).

Predictive variable	Mediation: KS		Mediation: KA		Dependent: RUK (OR)	
			
	M1		M2		M3	
			
	*B*	SE	*B*	SE	*B*	SE
Constant	0.35*	0.14	0.31*	0.12	3.73***	0.08
Control education^a^	−0.15*	0.06	−0.14*	0.06	0.01	0.03
Independent EX	−0.24***	0.04	0.05	0.04	0.08**	0.02
Moderation PS	0.14***	0.04	0.45***	0.04	0.07*	0.02
Interaction EX × PS	−0.15*	0.06	–0.01	0.02	0.01	0.01
Mediation						
KS					−0.05*	0.02
KA					0.05*	0.02
Interaction						
KS × PS					0.00	0.01
KA × PS					0.04	0.02
*R* ^2^	0.0592		0.245		0.1114	
*F*-test	9.8398***		50.6931***		9.7304***	

*N* = 630, *p < 0.05, **p < 0.01, ***p < 0.001.

^*a*^High school or below = 1, university = 2, graduate school or above = 3.

EX, experience; PS, political skill; KS, knowledge stickiness; KA, knowledge ambiguity; RUK, receipt of useful knowledge; OR, other-report.

Second, in model M2, the interaction between the independent variable experience and the moderation variable political skills (EX × PS) on the mediation variable knowledge ambiguity (KA) was not significant in either the self-reported (B = –0.01, *p* = 0.4859) or other-reported (B = –0.01, *p* = 0.4859) results. Thus, H5b was not supported.

**FIGURE 3:**
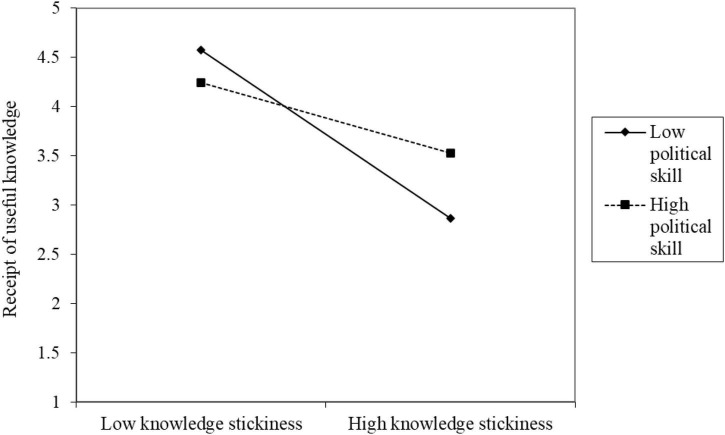
Diagram of the interaction of political skills affecting knowledge stickiness on receipt of useful knowledge.

Third, in model M3, the interaction between the mediation variable knowledge stickiness and the moderation variable political skills (KS × PS) on the dependent variable receipt of useful knowledge showed significant predictive power for self-reported (RUK[SR]; *B* = 0.04, *p* < 0.01) but not for other-reported (RUK[OR]; *B* = 0.00, *p* = 0.6437) results. Thus, H6a was supported. Figure 3 shows that under high knowledge stickiness, the receipt of useful knowledge is lower, whereas, under low knowledge stickiness, the receipt of useful knowledge is higher; the moderating effect of low political skills is stronger than that of high political skills.

**FIGURE 4:**
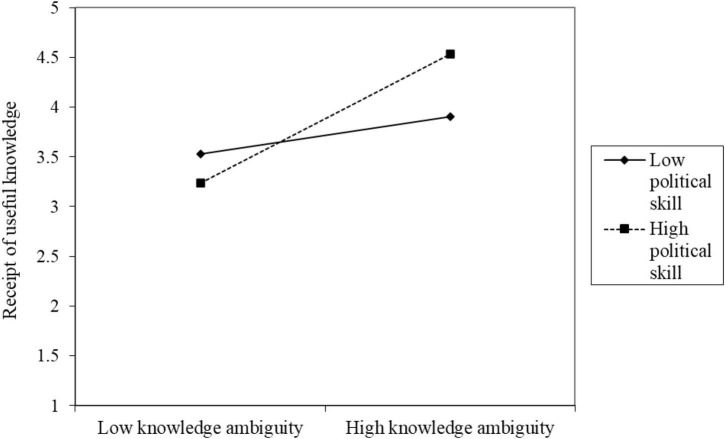
Diagram of the interaction of political skills affecting knowledge ambiguity on receipt of useful knowledge.

Fourth, in model M3, the interaction between the mediation variable knowledge ambiguity and the moderation variable political skills (KA × PS) on the dependent variable receipt of useful knowledge showed significant predictive power for self-reported (RUK[SR]; *B* = 0.18, *p* < 0.001) on other-reported (RUK[OR]; *B* = 0.05, *p* < 0.05) results. Thus, H6b was supported. Figure 4 shows that under high knowledge ambiguity, the receipt of useful knowledge is higher whereas under low knowledge ambiguity, the receipt of useful knowledge is lower; the moderating effect of high political skills is stronger than that of low political skills.

**FIGURE 5:**
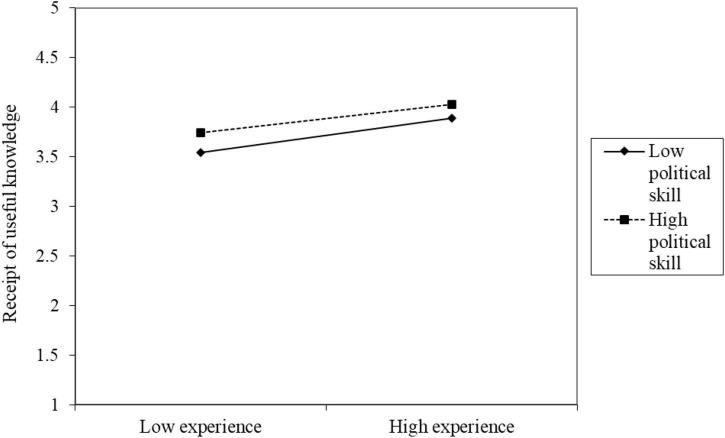
Diagram of the interaction of political skills affecting experience on receipt of useful knowledge.

Fifth, in model M3, the interaction between the independent variable experience and the moderation variable political skills (EX × PS) on the dependent variable receipt of useful knowledge showed significant predictive power for self-reported (RUK[SR]; *B* = –0.03, *p* < 0.05) but not for other-reported (RUK[OR]; *B* = 0.01, *p* = 0.5576) results. Thus, H7a was supported. Figure 5 shows that under high experience, the receipt of useful knowledge is higher whereas under low experience, the receipt of useful knowledge is lower; the moderating effect of high political skills is stronger than that of low political skills.

We tested moderating effect test of political skill. Table 5 presents the self-reported results while Table 6 summarizes the other-reported results. First, Model M1 also indicates that the interaction between the independent variable partner protectiveness and the moderation variable political skills (PP × PS) on the mediation variable KS was not significant in either the self-reported (B = 0.04, *p* = 0.1288) or other-reported (B = 0.04, *p* = 0.1288) results. Therefore, H5c was not supported.

**TABLE 5:** Analysis of the moderating effect of political skills to partner protectiveness on receiving useful knowledge (self-report).

Predictive variable	Mediation: KS		Mediation: KA		Dependent: RUK (SR)	
			
	M1		M2		M3	
			
	*B*	SE	*B*	SE	*B*	SE
Constant	0.30*	0.13	0.31*	0.12	3.72***	0.06
Control education^a^	−0.15*	0.06	−0.15*	0.06	–0.01	0.03
Independent PP	0.30***	0.03	–0.03	0.03	0.08***	0.02
Moderation PS	0.01	0.03	0.47***	0.03	0.23***	0.02
Interaction PP × PS	0.04	0.03	0.03	0.02	−0.04**	0.01
Mediation						
KS					−0.14***	0.02
KA					0.20***	0.02
Interaction						
KS × PS					0.08***	0.01
KA × PS					0.03*	0.01
*R* ^2^	0.1165		0.2411		0.4369	
*F*-test	20.5951***		50.4463***		60.2159***	

*N* = 630, *p < 0.05, **p < 0.01, ***p < 0.001.

^*a*^High school or below = 1, university = 2, graduate school or above = 3.

PP, partner protectiveness; PS, political skill; KS, knowledge stickiness; KA, knowledge ambiguity; RUK, receipt of useful knowledge; SR, self-report.

**TABLE 6:** Analysis of the moderating effect of political skills to partner protectiveness on receiving useful knowledge (other-report).

Predictive variable	Mediation: KS		Mediation: KA		Dependent: RUK (OR)	
			
	M1		M2		M3	
			
	*B*	SE	*B*	SE	*B*	SE
Constant	0.30*	0.13	0.31*	0.12	3.74***	0.08
Control education^a^	−0.15*	0.06	−0.15*	0.06	0.01	0.03
Independent PP	0.30***	0.03	–0.03	0.03	0.04	0.02
Moderation PS	0.01	0.03	0.47***	0.03	0.11***	0.02
Interaction PP × PS	0.04	0.03	0.03	0.02	–0.01	0.01
Mediation						
KS					−0.08***	0.02
KA					0.06*	0.02
Interaction						
KS × PS					0.01	0.01
KA × PS					0.04*	0.01
*R* ^2^	0.1165		0.2441		0.1013	
*F*-test	20.5951***		50.4463***		8.7538***	

*N* = 630, *p < 0.05, ***p < 0.001.

^*a*^High school or below = 1, university = 2, graduate school or above = 3.

PP, partner protectiveness; PS, political skill; KS, knowledge stickiness; KA, knowledge ambiguity; RUK, receipt of useful knowledge; OR, other-report.

Second, in model M2, the interaction between the independent variable partner protectiveness and the moderation variable political skills (PP × PS) on the mediation variable KA was not significant for the self-reported (B = 0.03, *p* = 0.2018) or other-reported (B = 0.03, *p* = 0.2018) results. Thus, H5d was not supported.

**FIGURE 6:**
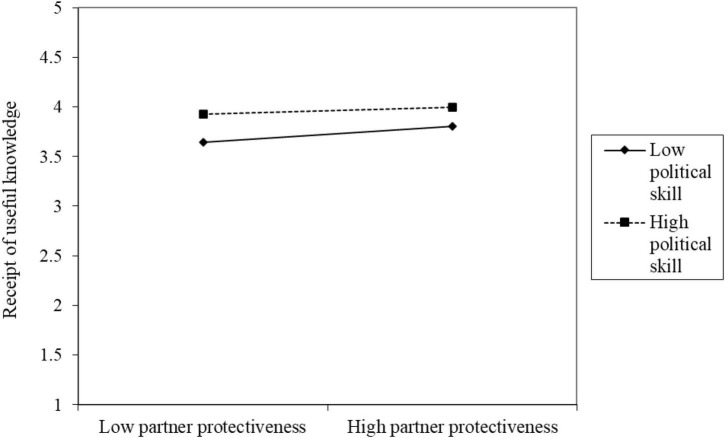
Diagram of the interaction of political skills affecting partner protectiveness on receipt of useful knowledge.

Third, in model M3, the interaction between the independent variable partner protectiveness and the moderation variable political skills (PP × PS) on the dependent variable receipt of useful knowledge showed significant predictive power for self-reported (RUK[SR]; *B* = –0.04, *p* < 0.001) but not for other-reported (RUK[OR]; *B* = –0.01, *p* = 0.5402) results. Thus, H7b was partially supported. Figure 6 shows that under high partner protectiveness, the receipt of useful knowledge is higher while under low partner protectiveness, the receipt of useful knowledge is lower; low political skills have a stronger moderating effect than high political skills.

### Hierarchical regression analysis

The regression analysis of experience on receipt of useful knowledge for both self-reported (EX→RUK[SR]; *B* = 0.17, *p* < 0.001) and other-reported results (EX→RUK[OR]; *B* = 0.08, *p* < 0.01) had a significant positive predictive effect (see Tables 3, 4). Therefore, H1a are supported. Meanwhile, the regression analysis of partner protectiveness on receipt of useful knowledge for self-reported results (PP→RUK[SR]; *B* = 0.08, *p* < 0.001) had a significant positive prediction effect, whereas, for other-reported results (PP→RUK[OR]; *B* = 0.04, *p* = 0.0635) did not (see Tables 5, 6). Therefore, H1b was not supported.

The regression analysis (*B* = –0.24, *p* < 0.001) of experience on knowledge stickiness (EX→KS) had a significant negative predictive effect, supporting H2a, whereas, the regression analysis (*B* = 0.05, *p* = 0.1649) of experience on knowledge ambiguity (EX→KA) was not significant, meaning H2b was not supported (see Tables 3, 4). The regression analysis (*B* = 0.30, *p* < 0.001) of partner protectiveness on knowledge stickiness (PP→KS) showed a significant positive predictive effect, supporting H2c, but the regression analysis (*B* = –0.03, *p* = 0.2918) of partner protectiveness on knowledge ambiguity (PP→KA) was not significant, so H2d was not supported (see Tables 5, 6).

The regression analysis of knowledge stickiness on received useful knowledge for the self-reported (KS→RUK[SR]; *B* = –0.08, *p* < 0.001) and other-reported (KS→RUK[OR]; *B* = –0.05, *p* < 0.05) results had a significant negative predictive effect, supporting H3a. Similarly, the regression analysis of knowledge ambiguity on received useful knowledge for self-reported (KA→RUK[SR]; *B* = 0.18, *p* < 0.001) and other-reported (KA→RUK[OR]; *B* = 0.05, *p* < 0.05) showed a significant positive predictive effect (see Tables 3, 4). Thus, H3b was not supported.

### Moderated mediation effect test of political skill

We tested moderating effect test of political skill. Table 7 summarize these results. First, When KS is a mediation variable, if is as low as M-1SD, the bootstrapping 95% CI test did not include zero (0.006, 0.041) for self-reported results (RUK[SR]). Thus, experience (EX) has low KS and has a significant conditional indirect effect on receipt of useful knowledge for self-reported results (RUK[SR]). However, for other-reported results (RUK[OR]), the bootstrapping 95% CI test (–0.001, 0.024) contained zero, indicating that EX has low knowledge stickiness and no significant conditional indirect effect on receipt of useful knowledge.

**TABLE 7:** Moderated mediation conditional indirect effect of experience on receiving useful knowledge.

Conditional indirect effect	Moderation: PS		*B*	SE	95% CI	
					
					Lower	Upper
EXperIRUK (SR)	–1 SD	2.916	0.022	0.009	0.006	0.041
	M	3.561	0.021	0.006	0.008	0.035
	±1 SD	4.207	011	0.007	–0.001	0.027
EX027SR)o (SR)	–1 SD	2.916	0.010	0.008	–0.002	0.030
	M	3.561	0.010	0.008	–0.004	0.028
	±1 SD	4.207	0.008	0.013	–0.018	0.036
EX036SR)o (OR)	–1 SD	2.916	0.010	0.006	–0.001	0.024
	M	3.561	0.013	0.006	0.001	0.025
	±1 SD	4.207	0.014	0.008	–0.002	0.033
EX033SR)o (OR)	–1 SD	2.916	0.001	0.003	–0.005	0.010
	M	3.561	0.003	0.003	–0.001	0.010
	±1 SD	4.207	0.003	0.006	–0.008	0.017

*N* = 630. Five thousand sets of samples are repeatedly selected for interval estimation under the 95% confidence interval of bootstrapping.

SD, standard deviation; M, mean; ±1 SD, indirect effect; CI, confidence interval. PS, political skill; EX, experience; KS, knowledge stickiness; KA, knowledge ambiguity; RUK, receipt of useful knowledge; SR, self-report; OR, other-report.

Second, When KS is as high as M + 1SD, the bootstrapping 95% CI test (–0.001, 0.027) contained zero for self-reported results (RUK[SR]), indicating that experience (EX) has high knowledge stickiness but no significant conditional indirect effect on receipt of useful knowledge. Other-reported results (RUK[OR]) produced similar results (bootstrapping 95% CI: –0.002, 0.033).

Third, When KA is a mediation variable and KS is as low as M-1SD, the bootstrapping 95% (CI) test for self-reported results (RUK[SR]; –0.002, 0.030) and other-reported results (RUK[OR]; –0.005, 0.010) contained zero, indicating that experience (EX) has low knowledge ambiguity, resulting in no significant conditional indirect effect on receipt of useful knowledge for either self-reported or other-reported results.

Fourth, when KA is a mediation variable and KS is as high as M + 1SD, the bootstrapping 95% CI test for self-reported results (RUK[SR]; –0.018, 0.036) and other-reported results (RUK[OR]; –0.008, 0.017) contained zero, indicating that experience (EX) has high knowledge ambiguity but no significant conditional indirect effect on receipt of useful knowledge for either self-reported or other-reported results. Based on these results, H8a was supported but H8b was not.

We tested moderating effect test of political skill. Table 8 summarize these results. First, When KS is a mediation variable and is as low as M-1SD, the bootstrapping 95% CI test for self-reported results (–0.090, –0.027) did not contain zero, suggesting that partner protectiveness (PP) has low knowledge stickiness and produces a significant conditional indirect effect on receipt of useful knowledge (self-reported [RUK]). However, for other-reported results, this same condition included zero in the bootstrapping 95% CI test (–0.048, –0.004), indicating that PP has low knowledge stickiness but produces no significant conditional indirect effect on receipt of useful knowledge (other-reported [RUK]).

**TABLE 8:** Moderated mediation conditional indirect effect of partner protectiveness on receiving useful knowledge.

Conditional indirect effect	Moderation: PS		*B*	SE	95% CI	
					
					Lower	Upper
PPperItio (SR)	–1 SD	2.916	–0.059	0.016	–0.090	–0.027
	M	3.561	–0.045	0.009	–0.063	–0.026
	±1 SD	4.207	–0.023	0.010	–0.046	–0.003
PP003Itio (SR)	–1 SD	2.916	–0.012	0.010	–0.035	0.004
	M	3.561	–0.008	0.007	–0.024	0.007
	±1 SD	4.207	–0.000	0.010	–0.020	0.020
PP020Itio (OR)	–1 SD	2.916	–0.024	0.011	–0.048	–0.004
	M	3.561	–0.025	0.008	–0.042	–0.009
	±1 SD	4.207	–0.024	0.010	–0.046	–0.006
PP006Itio (OR)	–1 SD	2.916	–0.001	0.003	–0.010	0.004
	M	3.561	–0.002	0.002	–0.009	0.002
	±1 SD	4.207	–0.000	0.004	–0.101	0.009

*N* = 630. Five thousand sets of samples are repeatedly selected for interval estimation under the 95% confidence interval of bootstrapping.

SD, standard deviation; M, mean; ±1 SD, indirect effect; CI, confidence interval.

PS, political skill; PP, partner protectiveness; KS, knowledge stickiness; KA, knowledge ambiguity; RUK, receipt of useful knowledge; SR, self-report; OR, other-report.

Second, when KS is a mediation variable and is as high as M + 1SD, both self-reported (RUK[SR]) and other-reported (RUK[OR]) results showed that PP has high knowledge stickiness, generating a significant conditional indirect effect on receipt of useful knowledge (bootstrapping 95% CI for self-reported: –0.046, –0.003; for other-reported: –0.046, –0.006).

Third, when KA is a mediation variable and KS is as low as M-1SD, PP has low knowledge ambiguity, producing no significant conditional indirect effect on receipt of useful knowledge for both self-reported (RUK[SR]) and other-reported (RUK[OR]) data (bootstrapping 95% CI test was –0.035, 0.004 and –0.010, 0.002).

Fourth, when KA is a mediation variable and KS is as low as M-1SD, PP has low knowledge ambiguity and shows no significant conditional indirect effect on receipt of useful knowledge for self-reported (RUK[SR]) results (bootstrapping 95% CI: –0.020, 0.020). Other-reported (RUK[OR]) results produced similar findings (bootstrapping 95% CI: –0.101, 0.009). Thus, based on these results, H8c were supported, but H8d was not.

## Discussion

This study explores the influence mechanism of characteristics of the source experience of the knowledge on the knowledge transfer performance. And the role of political skill and knowledge barriers was incorporated into this study. Through a series quantitative analysis, the finding demonstrates that the characteristics of the knowledge source will affect the performance of knowledge transfer. And the knowledge barriers will mediate the relationship between the characteristics of the knowledge source and the performance of knowledge transfer.

Meanwhile, the political skills of the knowledge source have an effect of moderation on the relationship between the characteristics of the knowledge source and the knowledge barriers. And the political skills of the knowledge source also have an effect of moderation on the relationship between the knowledge barriers and the performance of knowledge transfer. Also, the political skills of the knowledge source have an effect of moderation on the relationship between the characteristics of the knowledge source and the performance of knowledge transfer. Finally, the political skills of the knowledge source have the moderated mediation on the relationship among characteristics of the source of knowledge, knowledge barriers, and knowledge transfer performance.

### Theoretical implication

This study makes several theoretical contributions. First, this empirical research has demonstrated the importance of political skills in the relationship between knowledge source and recipient knowledge transfer performance. If an external source of knowledge possesses political skills and knows how to show sincerity, social interaction, interpersonal interaction, and impression management, they can adjust their behavior according to different situations and can reduce barriers to knowledge transfer as well as affect knowledge transfer performance. This research fills in the existing gaps in the literature related to the political skills of those who lack the source of knowledge in the knowledge transfer process.

Second, this study paves several meaningful directions for future study. On the one hand, an interactive environment between external knowledge sources and organizations, and firms can broaden their internal knowledge base from external knowledge resources, so how these external knowledge resources are successfully transformed into a firm’s unique competitive advantage and what variables are the key factors affecting the effectiveness of knowledge transfer in this interactive process are interesting topics that will continue to be sought and answered in future research. On the other hand, the characteristics of knowledge sources and recipients, such as the firm size gap, autonomy, and similarities and differences in technology and experience (Castro-Casal and Neira-Fontela, 2005; Aribou, 2013), will both affect the performance of knowledge transfer (Junni et al., 2012). Determining whether a difference exists in the performance of partner protectiveness between external knowledge sources and internal knowledge sources in knowledge transfer is a topic worthy of future research and discussion.

Third, in the area of knowledge management, Hansen et al. (1999) asserted that the choice between codification and personalization is the central one facing virtually all companies, and 80% of their knowledge sharing follows one strategy, 20% the other, and executives who try to excel at both strategies risk failing at both. Although the advice of (Hansen et al., 1999) is useful for deciding upon an initial strategic direction and setting suitable priorities, as a result of the intertwined nature of knowledge processes, organizations may find it necessary to evolve their knowledge strategy mix over time and augmenting the alternate strategy (Scheepers et al., 2004). Also, Venkitachalam and Willmott (2016) suggests that the combination of multi-operational types and four elements (i.e., competition, organizational size, organizational structure, and information technology) are highly relevant for determining the shifts between codification and personalization strategies in organizations. That is, the organization’s knowledge management strategy should be dynamic, and the choice of explicit knowledge and tacit knowledge should be flexible.

However, how does an organization dynamically adjust its knowledge management strategy to its development needs and business models at different stages? There is a fragmented perspective in terms of the range of whole knowledge management (Dwivedi et al., 2011). For this reason, the future study would provide more clear explanation as for these questions. Moreover, Hansen et al. (1999) mentioned that knowledge is closely tied to the person who developed it and is shared mainly through direct person-to-person contacts. In this process, the human networks characterized by sharing and flexibility have a large impact on knowledge transfer (Vãtãmãnescu et al., 2020; Lardón-López et al., 2022). As pointed out by Becerra et al. (2008), in a learning alliance, trust between partners has positive effect on the transfer of tacit knowledge. Finally, Hansen et al. (1999) have pointed out that computer technology plays an important role in the storage and transfer of explicit knowledge. In particular, today’s business landscape is increasingly characterized by the pervasive role of digital technologies, but the impact of digital technologies on knowledge transfer has not been widely discussed. In view of this, the four potential knowledge management research directions related to digitalization pointed out by Venkitachalam and Schiuma (2022) are worthy of further exploration by scholars.

Fourth, follow-up research can explore how external knowledge resources can be interfered by the organizational level (codification strategy) and individual level (personalization strategy) of the knowledge management strategy of external consultants (Hansen et al., 1999) through analyzing the moderating effect of knowledge barriers that influence organizational performance and individual performance. Finally, the effectiveness or usefulness of knowledge can only be measured when translated to organizational performance. Future studies may extend the model to examine the organization’s performance as a result of such knowledge transfer.

Third, the results of this study support the channel model proposition that organizations only need to remove barriers (including environmental barriers and transmission barriers) in the knowledge transfer process in order to ensure effective knowledge transfer (Szulanski, 1996; Simonin, 1999a,b; Sheng et al., 2013; Ciabuschi et al., 2017; Sikombe and Phiri, 2019). The results also support the embeddedness model proposition that organizations and other organizations are systematically engaged in interactive learning through an embeddedness institutional environment. Only through interpersonal interaction and participation in the daily work of the community can organizational members effectively transfer knowledge (Pousa and Mathieu, 2015).

### Practical implications

According to this research, if the person serving as a source of knowledge can make good use of political skills and demonstrate sincerity, social interaction, interpersonal interaction, and impression management, the individual can act as an objective third party in the organization and company, adjusting their behavior according to different work situations. Such individuals can also handle various relationships in the organizational hierarchy, serve as a communication and coordination bridge in all aspects, more effectively influence others, and positively affect the performance of knowledge transfer.

The management implications of this study focus on three points. First, external knowledge sources can become external resources of the firm organization through the key knowledge they possess. Knowledge is a resource that can generate dependencies, so it may become a source of power. If the knowledge source has the key knowledge resources needed by the firm, can provide the organization with capabilities that cannot be generated internally, and has the ability to control these important resources, it can impact the behavior of the dependent firms and enable the external knowledge transfer of resources and of exchange economic benefits with the firms.

Second, it is necessary for the external knowledge source to act as an external medium in an open system of organizational input and output, and there is an interactive relationship between the firm organization. The development process of the firm organization will inevitably produce operating bottlenecks at different stages. Firms can learn and acquire knowledge resources from external knowledge sources, integrate and transform them into new internal knowledge, apply new knowledge to products and services, and obtain the value of the firm’s key competitiveness.

Finally, organizations need to survive by acquiring resources in the environment. No organization is self-sufficient, and every organization must engage in exchanges with the environment. During the development of a firm’s organization, it must continually broaden its horizons and make full use of society’s extensive external resources. No matter how many internal resources a firm has, it is still limited. In addition to having the internal resources of the organization itself, the firms must also have the ability to utilize external resources. If a firm can make good use of the knowledge and technology of external knowledge sources to transfer to its employees, then its employees can contribute the knowledge they have learned to the firm’s success.

## Data availability statement

The original contributions presented in this study are included in the article/supplementary material, further inquiries can be directed to the corresponding author.

## Author contributions

S-LL and T-KH designed the study, performed the statistical analysis, formulated the conclusion, and wrote the manuscript. MT conducted the research and revised the manuscript. All authors contributed to the article and approved the submitted version.
